# Highly versatile cancer photoimmunotherapy using photosensitizer-conjugated avidin and biotin-conjugated targeting antibodies

**DOI:** 10.1186/s12935-019-1034-4

**Published:** 2019-11-15

**Authors:** Naoto Shirasu, Hirotomo Shibaguchi, Hiromi Yamada, Masahide Kuroki, Shin’ichiro Yasunaga

**Affiliations:** Department of Biochemistry, Faculty of Medicine, Fukuoka University, 7-45-1 Jonan-ku, Fukuoka, 814-0180 Japan

**Keywords:** Avidin, Biotinylated antibody, Cancer stem cell, Tumor microenvironment, Photoimmunotherapy

## Abstract

**Background:**

Photoimmunotherapy (PIT) employing antibody-photosensitizer conjugates is a promising treatment for cancer. However, the fixed antigen specificity severely limits the efficacy and the applicability. Here we describe a universal strategy for PIT of cancer by using a near-infrared (NIR) photosensitizer IRDye700DX-conjugated NeutrAvidin, designated as AvIR, together with various biotinylated antibodies (BioAbs) for cellular targeting.

**Methods:**

Cytotoxicity of AvIR-mediated PIT was evaluated by fluorescence imaging and cell viability assay. Phototoxic effect on tumorigenicity was assessed by tumorsphere-formation assay and Matrigel invasion assay. Cancer stem cell-like side-population (SP) cells were identified by flow cytometry.

**Results:**

CHO cells stably expressing carcinoembryonic antigen or EpCAM were pre-labeled with each BioAb for the corresponding antigen, followed by AvIR administration. NIR light irradiation specifically killed the targeted cells, but not off-targets, demonstrating that the AvIR-mediated PIT does work as expected. CSC-like subpopulation of MCF-7 cells (CD24^low^/CD44^high^) and SP of HuH-7 cells (CD133^+^/EpCAM^+^) were effectively targeted and photokilled by AvIR-PIT with anti-CD44 BioAb or anti-CD133/anti-EpCAM BioAbs, respectively. As results, the neoplastic features of the cell lines were sufficiently suppressed. Cancer-associated fibroblast (CAF)-targeted AvIR-PIT by using anti-fibroblast activation protein BioAb showed an abolishment of CAF-enhanced clonogenicity of MCF-7 cells.

**Conclusions:**

Collectively, our results demonstrate that AvIR-mediated PIT can greatly broaden the applicable range of target specificity, with feasibility of efficacious and integrative control of CSC and its microenvironment.

## Background

**Fig. 1:**
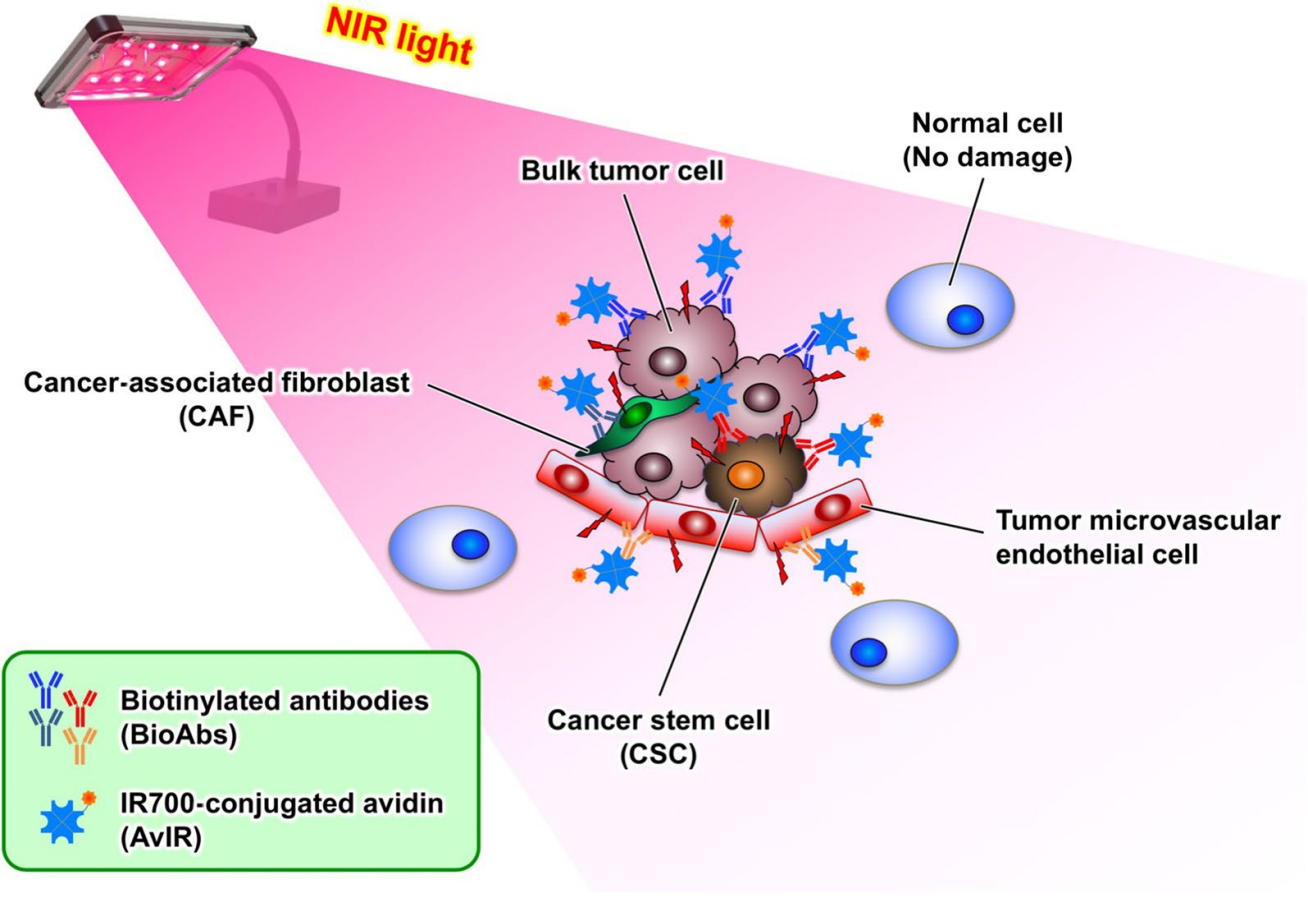
Schematic representations of AvIR-mediated PIT. Due to the cellular targeting by BioAb(s) specific to the tumor cells and/or tumor-supporting cells, AvIR can exert the phototoxicity only on the targeted cells upon NIR irradiation, without any damage to normal tissues. As long as cell type-specific BioAbs are available, potential therapeutic target cells of AvIR-PIT are virtually unlimited, allowing the highly integrated tumor control

Photoimmunotherapy (PIT), which is a targeted photodynamic therapy using a photosensitizer (PS)-loaded monoclonal antibody (mAb) specific for tumor-associated antigen (TAA), has been developed as a safe and an attractive therapeutic modality for cancer (reviewed in [1, 2]). With excitable light irradiation, PIT exerts a remarkable cytotoxicity against only tumor cells targeted by PS-mAb conjugates. Near-infrared (NIR) phthalocyanine dye, IRDye700DX (IR700), has been accepted to have promising PS moiety of the PIT agents, because of its excitation wavelength (690 nm) with high tissue-permeability and of the photochemical property to induce strong cytotoxicity only when the conjugate bound to the plasma membranes of the target cells is exposed by NIR light [3, 4]. Indeed, to date, IR700 have been successfully applied to several PIT utilizing mAbs against clinically relevant TAAs, such as carcinoembryonic antigen (CEA) [5], human epidermal growth factor receptor 2 (HER2) [6, 7], and epidermal growth factor receptor (EGFR) [8, 9]. Phase III clinical trial of PIT with an ASP-1929 (anti-EGFR cetuximab-IR700 conjugate) in patients with recurrent head and neck cancer is currently underway across countries (ClinicalTrials.gov Identifier: NCT03769506). More recently, the target of IR700-mediated PIT has been expanded to the intra-/peri-tumoral non-neoplastic cells that serve to support and maintain the tumor microenvironment. These cells include, for example, cancer-associated fibroblasts (CAFs) [10], which are important constituents of the tumor stroma, and vascular endothelial cells that construct tumor neovasculature [11]. Thus, IR700-mediated PIT has great potential to be an extensively applicable cancer therapy. However, solid tumors are generally composed of heterogeneous cell populations, which could arise from cancer stem cells (CSCs) [12], and it is well known that the expression pattern of TAAs and the organization of the tumor microenvironment often change dynamically depending on the malignant progression and the course of radiotherapy and chemotherapy [13]. In addition, tumors can acquire resistance to single-agent therapy in many instances. Therefore, the current cancer-targeted therapies involving PIT which utilize a mAb against a single TAA alone are considered to be highly difficult to cure cancer, even if temporary tumor regression is achieved. In order to effectively apply the IR700-PIT to a broad range of cancer types and of changes in TAA expression, it is considered necessary to prepare a panel of IR700-mAb conjugates with different specificity corresponding to various target TAAs on a case-by-case basis; however, such approach is extremely complicated, costly in terms of time and money, and unrealistic. To overcome these problems and realize a highly versatile PIT applicable to various cancers and tumor-supporting cells, we aimed to develop a novel PIT utilizing IR700-conjugated NeutrAvidin, designated as AvIR, in combination with biotinylated antibodies (BioAbs) for cell-specific targeting. In this strategy, target cells are pre-labeled with single or multiple BioAbs specific to cell surface marker(s), followed by binding AvIR exclusively to them owing to the tremendous affinity and specificity to biotin, then NIR irradiation is applied for photokilling of the targeted cells (Fig. 1). Myriad of BioAbs, whether commercially and clinically available or in-house developed, can dramatically expand the applicability of conventional PIT, allowing the unlimited target specificity without repetitive preparation of PS-mAb conjugates. If AvIR-mediated PIT works effectively, the sequential or simultaneous use of various BioAbs would be achievable a universal PIT capable of responding to altered expression of TAAs, enabling comprehensive cancer therapy that targets not only heterogeneous tumor cell populations including CSCs that express different TAAs, but also stromal and vascular endothelial cells that constitute the tumor microenvironment.

## Materials and methods

### Cell lines

Luciferase-expressing cell lines derived from human gastric adenocarcinoma (MKN-45), breast adenocarcinoma (MCF-7) and hepatocellular carcinoma (HuH-7), were obtained from the Japanese Collection of Research Bioresources (Osaka, Japan). MKN-45 cells were maintained in RPMI1640 Glutamax medium (Thermo Fisher Scientific, Tokyo, Japan) supplemented with 10% fetal bovine serum (FBS; Equitech-Bio, Kerrville, TX) in an atmosphere of 5% CO_2_ at 37 °C. MCF-7 and HuH-7 cells were maintained in Dulbecco’s minimal essential medium (DMEM; Thermo Fisher Scientific) instead of RPMI1640. Two Chinese hamster ovary (CHO) cell lines, human CEA-expressing CHO-CEA and human EpCAM-expressing CHO-EpCAM cells, have been described previously [5], and were cultured in α-modified minimum essential medium (α-MEM; Thermo Fisher Scientific) supplemented with 10% FBS and 2 mM glutamine. Murine tumor endothelial cell line 2H-11 was obtained from the American Type Culture Collection (Manassas, VA) and was maintained in DMEM supplemented with 10% FBS and 2 mM glutamine (Wako Pure Chemicals, Osaka, Japan). Primary human breast CAF derived from an infiltrating ductal-carcinoma tissue was purchased from Asterand (Detroit, MI) and maintained in DMEM supplemented with 10% FBS and penicillin–streptomycin.

### Antibodies

Fully human mAbs to human CEA (clone: C2-45) or human EpCAM (clone: M13-57) were previously established in our laboratory [14, 15]. Anti-human CD44 (clone F10-44-2) was purchased from Abcam (Cambridge, MA). Anti-human CD24 (clone SN3) was purchased from Thermo Fisher Scientific. Anti-human fibroblast activation protein (FAP) polyclonal antibody was purchased from R&D Systems (Minneapolis, MN). Anti-mouse CD105 (clone MJ7/18) were purchased from BioLegend (Tokyo, Japan). Biotinylated anti-human CD133/1 (hereinafter “Bio-CD133”; clone AC133) was purchased from Miltenyi Biotec (Bergisch-Gladbach, Germany).

### Biotinylation of antibodies

BioAbs, except for Bio-CD133, for AvIR-mediated PIT were chemically prepared using EZ-Link Sulfo-LC-NHS-Biotinylation Kit (Thermo Fisher Scientific) following manufacturer’s instructions. A Zeba Desalt Spin Column (Thermo Fisher Scientific) was used to remove excess biotin reagent and exchange the buffer with Dulbecco’s phosphate-buffered saline (DPBS). We also performed a biotinylation of human immunoglobulin G (IgG) for using as an irrelevant control (Bio-hIgG).

### Preparation of AvIR

NeutrAvidin (2 mg) (Thermo Fisher Scientific) was incubated with IRDye700DX *N*-hydroxysuccinimide ester (100 µg) (LI-COR Biosciences, Lincoln, NE) in 2 ml of 100 mM sodium phosphate buffer (pH 9.0) for 2 h at room temperature. The reaction mixture was applied onto a Zeba desalting column to purify the AvIR. The concentration of AvIR and the dye/protein ratio was spectroscopically determined by measuring the absorbance at 280 nm and 689 nm, and by using the following molar extinction coefficients (ε): 101,640 M^−1^ cm^−1^ for NeutrAvidin at 280 nm [16], 165,000 M^−1^ cm^−1^ for IR700 at 689 nm. The dye/antibody ratio of AvIR was typically ~ 2.2.

### Fluorescence analysis of phototoxicity induced by AvIR-PIT

The phototoxic effect of AvIR was assessed by using the LIVE/DEAD Cell Imaging Kit (Thermo Fisher Scientific). Cells were seeded onto an 8-well Lab-Tek II chamber slide (Thermo Fisher Scientific) at a density of 10,000 cells/well 1 day before AvIR-PIT. The next day, the cells were treated by adding biotinylated anti-CEA (Bio-CEA), biotinylated anti-EpCAM (Bio-EpCAM) (5 µg/ml), or an equal volume of DPBS for 30 min, followed by adding AvIR (5 µg/ml) with another incubation for 30 min. The cells were exposed to NIR light (3 J/cm^2^) from a light-emitting diode (LED) light source (Shiokaze Giken, Niigata, Japan), which emits red light with a peak at 690 nm. The irradiation energy density was measured with a PM100D optical power meter (Thorlabs, Tokyo, Japan). The irradiated cells were incubated with a mixture of Live Green and Dead Red solutions for 20 min and were subsequently imaged using a fluorescence microscope BZ-9000 (Keyence, Osaka, Japan). To assess the target specificity, AvIR-mediated PIT was also performed for co-cultured CHO-CEA and CHO-EpCAM cells. The CHO-CEA cells were stained with CellTracker Blue dye (Thermo Fisher Scientific) and were co-cultured with unlabeled CHO-EpCAM cells in a Lab-Tek II chamber on the day before AvIR-PIT.

### Quantitative evaluation of phototoxicity

The PIT-induced changes in cellular viability were assessed by using the CellTiter-Glo assay (Promega, Madison, WI). Briefly, the cells were plated onto a white-walled 96-well plate (Thermo Fisher Scientific) at 10,000 cells/well and were cultured overnight. On the following day, BioAb was added to the wells at the indicated concentrations with incubation for 30 min. Then, AvIR was added to the wells at the indicated concentrations. After another 30 min incubation, the cells were irradiated with NIR light (3 J/cm^2^). After irradiation, an aliquot of CellTiter-Glo reagent was added into each well, and the plate was shaken for 2 min. The plate was, then, incubated for 10 min at room temperature, and the luminescence was measured on a TriStar LB 941 multimode reader (Berthold Technologies, Bad Wildbad, Germany).

### Flow cytometry

To examine the binding characteristics of AvIR, CHO cells were labeled with Bio-CEA or Bio-EpCAM for 30 min and were stained with AvIR for 30 min. The cells were analyzed by fluorescence-activated cell sorting (FACS) using FACS Calibur or FACS Aria Fusion (BD Biosciences, Sunnyvale, CA) with FCS Express 6 software (De Novo Software, Los Angeles, CA). The following monoclonal anti-human antibodies were used for flow cytometric immunophenotyping of MKN-45, MCF-7, and HuH-7 cells: CD24-PE (phycoerythrin), CD44-FITC (fluorescein isothiocyanate), CD44-PE, CD133-PE, CEA-FITC, and EpCAM-AlexaFluor 488. All of them were purchased from BioLegend except for CD133-PE, purchased from Miltenyi Biotec. BioAb-labeled cells were stained with AvIR or streptavidin-PerCP-Cy5.5 (BD Biosciences). AvIR was excited with ultraviolet light at 375 nm, a wavelength within the minor absorption range of IR 700, and the fluorescence emission was measured with 670 LP filters.

### Tumorsphere-formation assay

Tumorsphere assay on MCF-7 cells, derived from breast cancer, was performed using MammoCult medium (Stem Cell Technologies, Vancouver, BC, Canada) with serum replacement, hydrocortisone, heparin, and antibiotics according to manufacturer’s instructions. Briefly, CD24^low^/CD44^high^ CSC subpopulation of MCF-7 cells were enriched and obtained by twice-repeated negative selections using the EasySep PE selection kit (Stem Cell Technologies) with CD24-PE followed by a positive selection using the EasySep FITC selection kit (Stem Cell Technologies) with CD44-FITC. The magnetically sorted MCF-7 cells or unsorted bulk cells were seeded onto a 6-well Ultra-low Attachment culture plate (Corning, NY) with 2 ml of complete MammoCult medium at a cell density of 5000 cells/ml. Then, this was incubated for 7 days at 37 °C in a humidified atmosphere containing 5% CO_2_. The resulting tumorspheres (> 60 µm) were counted by visual inspection in light microscopy. To investigate the phototoxic effect of AvIR on the clonogenicity, MCF-7 cells were PIT-treated with the indicated BioAb and AvIR (5 µg/ml each), and then the dead cells were removed by using ClioCell magnetic nanoparticles (ClioCell; London, UK). The resultant live cells were assessed for sphere-formation as above.

In the case of HuH-7 cells, derived from hepatocellular carcinoma, the sphere-formation capacity was determined using Cancer Stem Cell Medium (PromoCell, Heidelberg, Germany). HuH-7 cells were FACS-sorted into 4 subpopulations according to the immunophenotype regarding the expression of CD133 and EpCAM using FACSAria Fusion cell sorter. The sorted cells were seeded onto an Ultra-low Attachment plate at a density of 2000 cells/well. The cells were incubated for 10 days in a 5% CO_2_ atmosphere at 37 °C. The number of tumorspheres (> 100 µm) was manually counted.

### Matrigel invasion assay

Cell invasion was assayed using the CytoSelect 24-well Cell Migration and Invasion Assay according to the manufacturer’s instruction (Cell Biolabs, San Diego, CA). In brief, MCF-7 cells were resuspended in serum-free DMEM containing 0.1% bovine serum albumin. The cell suspension (1 × 10^6^ cells/ml) was added to the top insert, whereas DMEM containing 10% FBS was added to the bottom chamber. The cells were incubated at 37 °C for 24 h, and the insert was transferred to a well containing Cell Stain Solution. After incubation for 10 min, the stained insert was washed and air-dried. The migratory cells were counted with a light microscope.

### Analysis of side-population fraction in HuH-7 cells

Side-population (SP) analysis was basically performed according to the protocol by Goodell et al. [17] with some modifications. HuH-7 cells were dissociated into single cells with Accutase (MS TechnoSystems, Osaka, Japan), washed with DPBS, resuspended in pre-warmed DMEM supplemented with 2% FBS and 10 mM HEPES (4-(2-hydroxyethyl)-1-piperazineethanesulfonic acid) (Sigma, St. Louis, MO) at a density of 1 × 10^6^ cells/ml. Then, Hoechst 33342 dye (Dojindo Laboratories, Kumamoto, Japan) was added to the cells at a final concentration of 5 μg/ml. The cells were incubated in 37 °C water bath for 120 min in the presence or absence of an ATP-binding cassette transporter inhibitor verapamil (50 μg/ml) (Wako Pure Chemicals). Following incubation, the cells were washed with ice-cold Hanks balanced salt solution (HBSS) (Wako Pure Chemicals) with 2% FBS and 10 mM HEPES. The cells were filtered through a 40 μm nylon mesh to obtain a single cell suspension and kept at 4 °C in the dark until flow cytometric analysis using FACSAria Fusion. Hoechst 33342 was excited with ultraviolet light at 375 nm and fluorescence emission was measured with 450/20 (Hoechst blue) and 670 LP (Hoechst red) optical filters. During the FACS analysis, dead cells were excluded by using the viability dye SYTOX AADvanced (Thermo Fisher Scientific). To investigate the effect of AvIR-mediated PIT on SP fraction, HuH-7 cells were pre-labeled with Bio-CD133 and Bio-EpCAM (2.5 µg/ml each) for 30 min and incubated with adding 5 µg/ml AvIR for another 30 min. The cells were irradiated with NIR light (3 J/cm^2^), and the dead cells were removed by ClioCell treatment. The live cells were cultured under standard condition for another 2 passages, and the SP analysis was performed as described above.

### Soft agar colony formation assay

For the evaluation of CAF-assisted clonogenicity, modified soft agar colony formation assay was performed. Briefly, primary human breast CAFs were seeded onto a well of 6-well plate at a density of 7 × 10^4^ cells/well and cultured overnight. The next day, the culture medium was removed, and molten 0.6 ml 0.8% DNA grade agarose in DMEM with 10% FBS was added to the well. After solidification, 0.8 ml of 0.4% soft agar in complete MammoCult medium containing 5 × 10^3^ MCF-7 cells was layered on the solidified base agarose. Then, 0.8 ml of complete MammoCult was added to the well, and the cells were cultured for 7 days. The medium was exchanged for complete MammoCult with or without biotinylated anti-FAP (Bio-FAP; 5 µg/ml). After incubation for 12 h, AvIR (5 µg/ml) or vehicle was added to the medium, followed by another incubation for 12 h. The plate was irradiated with NIR light (6 J/cm^2^) and returned back to the incubator for further culturing for 11 days. The colonies formed in soft agar layer was counted manually.

### AvIR-PIT treatment against tumor endothelium model

We used 2H-11 cells for the formation of tumor endothelial tubes. The tubes were prepared on the tumor-derived extracellular matrix gel in a well of 96-well plate by using the Endothelial Tube Formation Assay (Cell Biolabs). AvIR-mediated PIT (3 J/cm^2^) was performed against the 2H-11 tubes using biotinylated anti-CD105 (Bio-CD105) and AvIR (5 µg/ml each). After a gentle washing, LIVE/DEAD cell imaging was performed.

### Statistical analysis

The data are expressed as the mean ± standard error of the means (SEM) from a minimum of three experiments. Statistical significance was evaluated by Student’s *t*-test or one-way analysis of variance (ANOVA), followed by Dunnett’s or Tukey’s multiple-comparison test. All statistical analyses were done using the GraphPad Prism 8 (GraphPad Software, San Diego, CA). p-values < 0.05 were considered to be statistically significant.

## Results

### Antigen-specific phototoxicity induced by AvIR-PIT

**Fig. 2:**
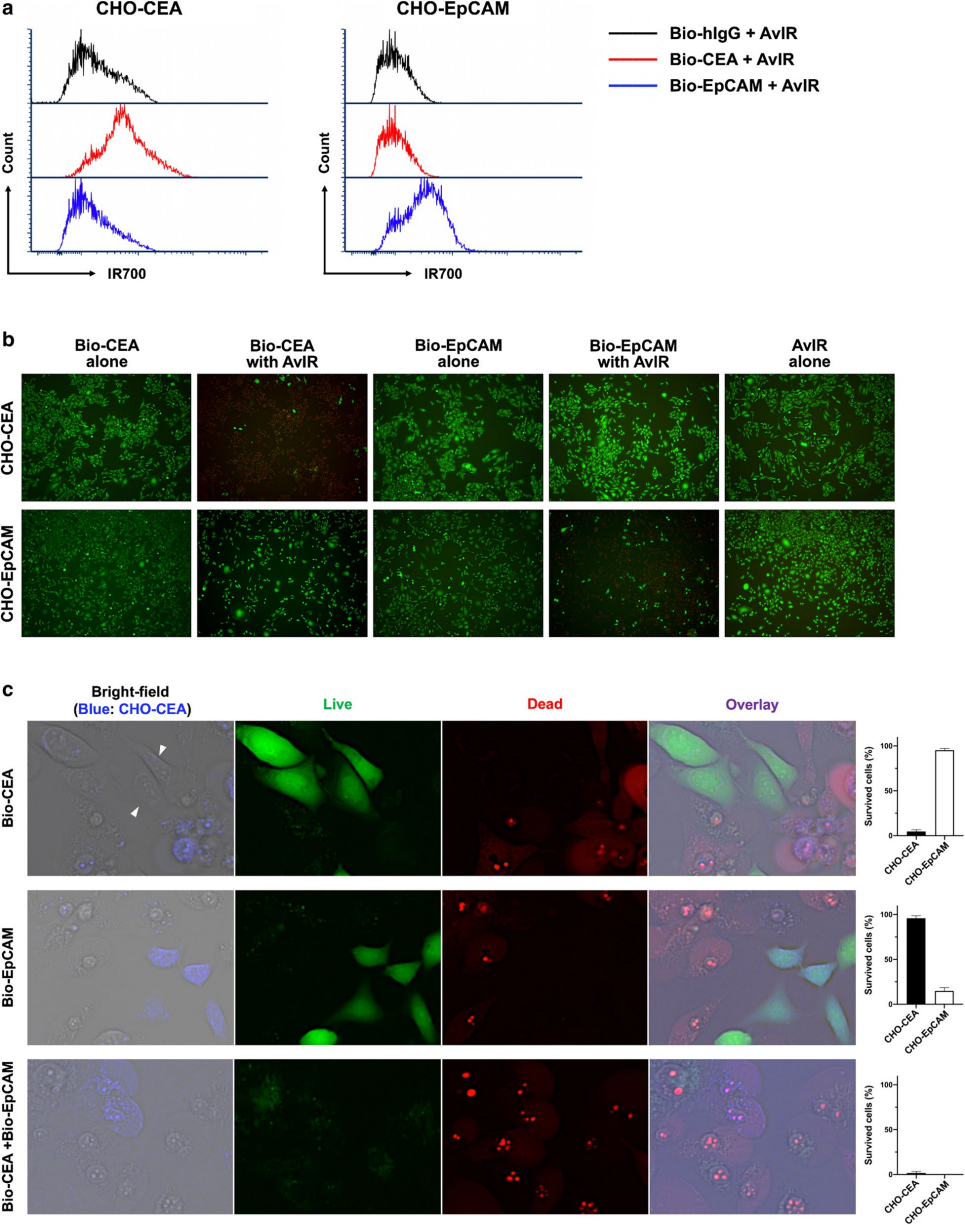
Target-specific phototoxicity of AvIR-mediated PIT. **a** TAA-specific binding of AvIR was assessed by flow cytometry. CHO cells were first incubated with unlabeled mAb or BioAb and then stained with AvIR. **b** CHO-CEA and CHO-EpCAM cells were incubated with the indicated BioAb (5 µg/ml) or DPBS for 30 min and further incubated with AvIR (5 µg/ml) for 30 min. Subsequently, the cells were irradiated with NIR light (3 J/cm^2^). After NIR light exposure, the cells were stained using LIVE/DEAD cell imaging kit, and the images were acquired using fluorescence microscope to determine whether they were alive (green) or dead (red). **c** LIVE/DEAD cell images of AvIR-PIT-treated co-culture of CHO-CEA and CHO-EpCAM cells. On the day before PIT treatment, the CHO-CEA cells were pre-stained with CellTracker Blue and then co-cultured with unstained CHO-EpCAM cells. The cells were targeted by incubation with the indicated BioAb(s), and AvIR-PIT was performed. The magenta cells in the overlaid images indicate dead CHO-CEA cells. Note that the CHO-EpCAM cells, even in contact with CHO-CEA cells (arrowheads in the bright-field image of top row) were alive. Data in the rightmost panels show the percentages of live cells that survived PIT-treatment measured by counting the green fluorescent cells on five randomly selected fields under the microscope. One hundred percent represents the total CHO-CEA or CHO-EpCAM cells per field. The data are the means ± SEM (n = 3)

In order to demonstrate the feasibility of AvIR based PIT, we first tested CHO cells stably expressing human CEA or EpCAM as a model of target tumor cells. Flowcytometric analysis showed that AvIR specifically bound to the cells pre-labeled with a BioAb (Bio-CEA or Bio-EpCAM) to the corresponding antigen (Fig. 2a). To explore the phototoxic effects of AvIR, we performed a LIVE/DEAD cell viability assay. We found that AvIR exerted strong antigen-specific cytotoxicity toward the BioAb-labeled CHO cells upon NIR irradiation (Fig. 2b). Even in the presence of AvIR in culture medium, no phototoxic effect was observed when unmatched BioAb was used for pre-labeling of the cells or when AvIR alone, without BioAb, was used for PIT. These results indicate that AvIR-mediated PIT can specifically kill the BioAb-targeted cells, and are consistent with the previous studies on PIT with IR700-mAb conjugates, in which the conjugates exert the phototoxicity only when bound to the cell membranes; however, it was clearly revealed that, to achieve such phototoxicity, IR700 does not necessarily have to be conjugated directly with targeting antibody molecules.

To further investigate the target-specificity of AvIR-mediated PIT, we carried out PIT experiment on CHO-EpCAM cells co-cultured with CellTracker-stained CHO-CEA cells, followed by LIVE/DEAD imaging. When co-cultured cells were pre-labeled with Bio-CEA, AvIR-PIT treatment selectively killed the CHO-CEA cells with no damage to any CHO-EpCAM cells even adjacent to ruptured CHO-CEA cells (Fig. 2c, top row). On the other hand, when Bio-EpCAM was used for pre-labeling, only the CHO-EpCAM cells were damaged (Fig. 2c, middle row). If both BioAbs were used, almost all of each kind of CHO cells was killed (Fig. 2c, bottom row). These results indicate that the phototoxicity of AvIR-mediated PIT is highly antigen-specific and again confirmed that membrane binding of AvIR via BioAb are requisite for evoking effective photocytotoxicity.

**Fig. 3:**
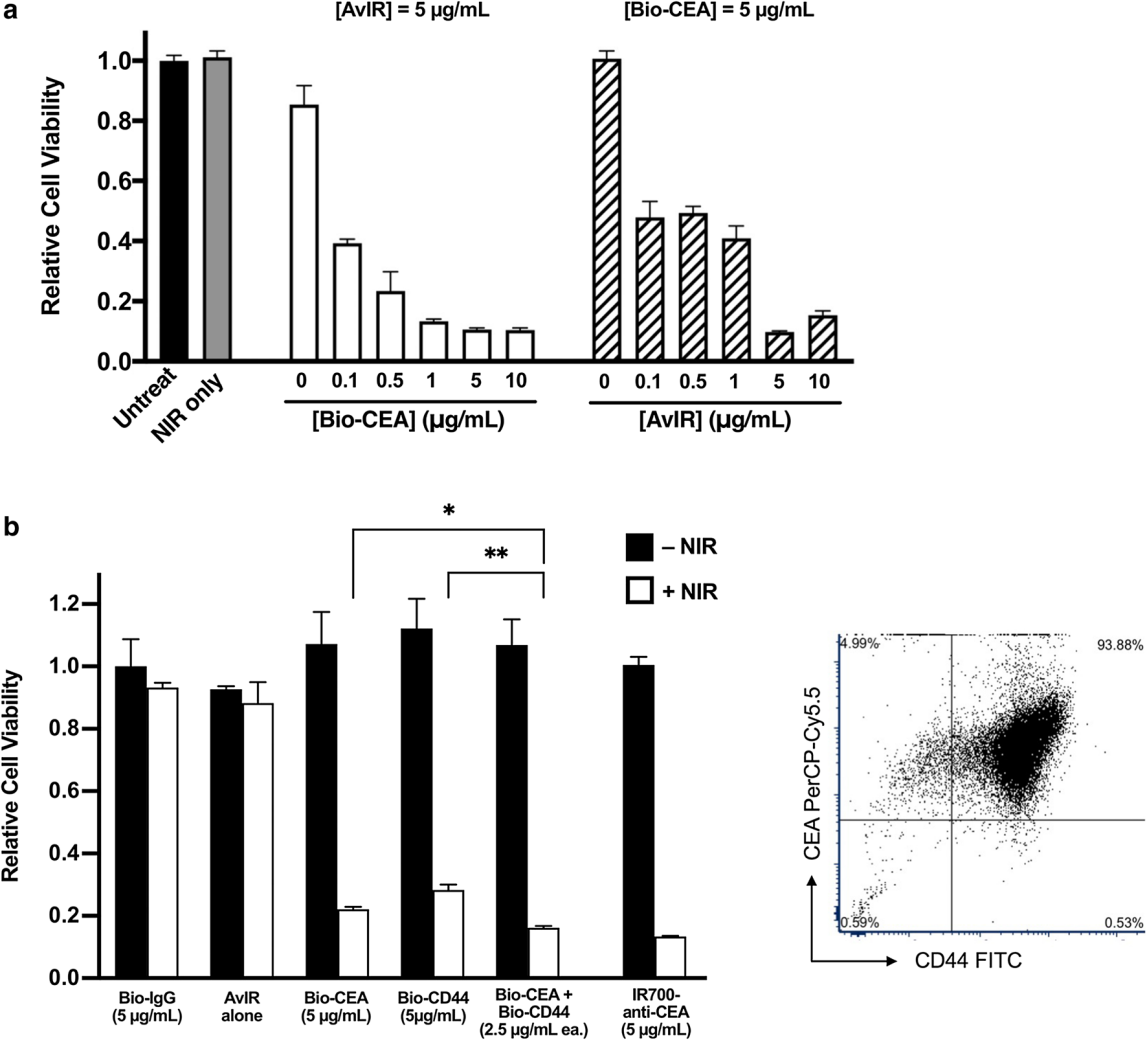
Effect on MKN-45 viability of AvIR-PIT. **a** The relative cell viability of MKN-45 cells was evaluated after AvIR-PIT treatment (3 J/cm^2^) with various concentrations of Bio-CEA or AvIR by using the CellTiter-Glo reagent. When the dose of Bio-CEA was variable, that of AvIR was constant, and vice versa. **b** The cell viability after AvIR-mediated PIT with each indicated BioAb was evaluated (left panel). When IR700-anti-CEA was used for cellular targeting, NIR light irradiation (3 J/cm^2^) was done without adding AvIR as conventional PIT treatment. The data are the means ± SEM (n = 3, *p < 0.05, **p < 0.01 vs. combination therapy using both Bio-CEA and Bio-CD44, one-way ANOVA with Dunnett’s test). Representative dot plot of the FACS analysis for the expression of CEA and CD44 is also shown (right panel)

In order to evaluate the phototoxic effect, the cell viability after AvIR-PIT was assessed with the CellTiter-Glo assay, which quantify ATP amount in the living cells. AvIR-PIT using Bio-CEA showed potent, agent-dose-dependent phototoxicity on MKN-45 cells positive for CEA (Fig. 3a). In all the following experiments, unless otherwise specified, we used BioAb and AvIR at 5 µg/ml, respectively. MKN-45 cells express CD44 as well as CEA, and indeed, Bio-CD44 was also found to be an effective MKN-45-targeting antibody for AvIR-PIT (Fig. 3b). Coadministration of Bio-CEA and Bio-CD44 (2.5 µg/ml each) showed improved phototoxicity compared with either BioAb (5 µg/ml) alone. Of note, the phototoxic effect of AvIR-PIT with Bio-CEA was not so inferior to that of conventional PIT using IR700-conjugated anti-human CEA mAb, as we previously reported [5], again suggesting that indirect IR700-labeling of the target cells is not particularly detrimental to the efficacy of PIT.

### AvIR-mediated PIT targeting CSCs

**Fig. 4:**
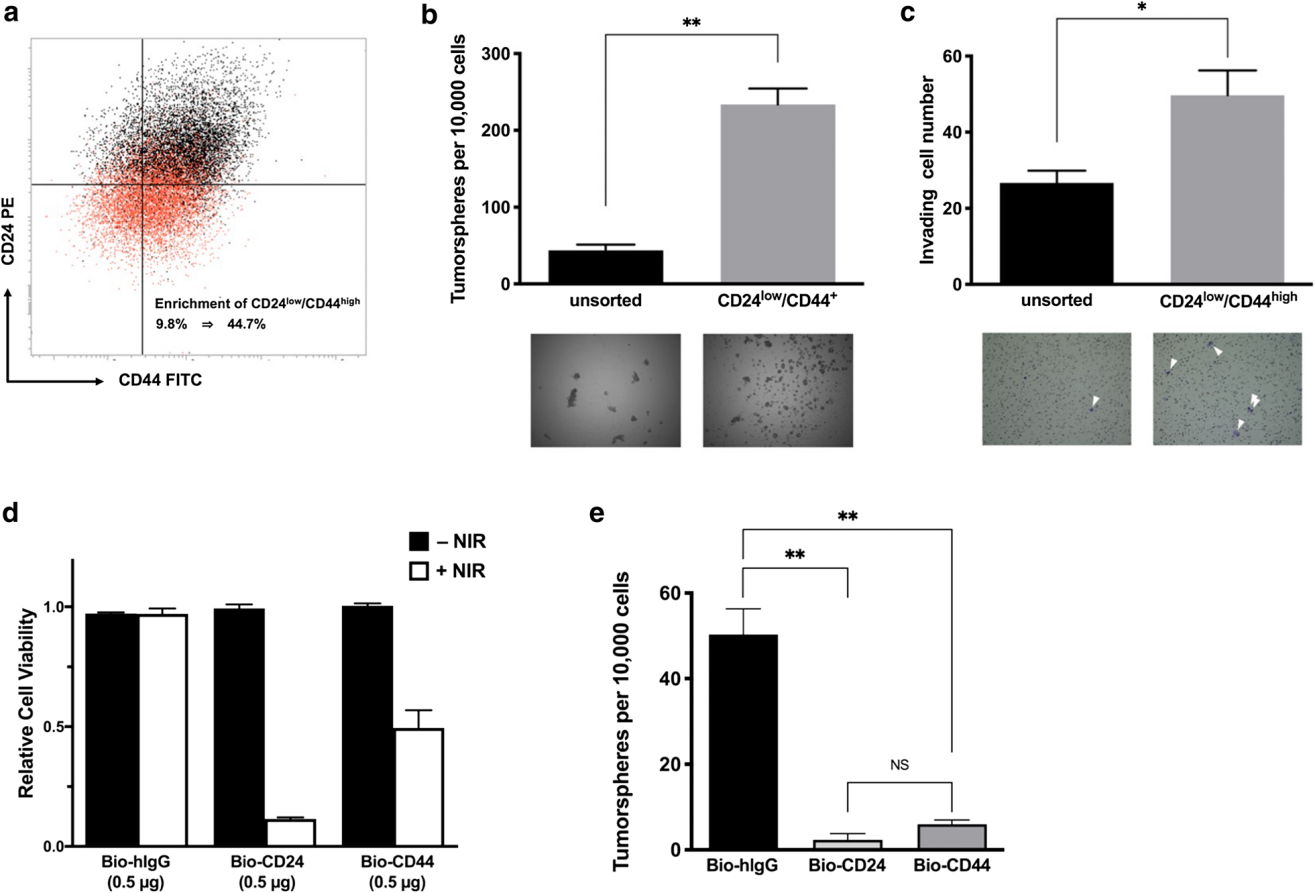
Phototoxic effect on CSC-like subpopulation of MCF-7 cells induced by AvIR-PIT. **a** Representative dot plot derived from flow cytometry analysis examining the expression of cell surface CD24 and CD44 of MCF-7 cells. Black dots indicate the unsorted cells, while red dots represent the cells after magnetic cell sorting to enrich the CD24^low^/CD44^+^ population. **b**, **c** Tumorsphere-formation assay and Matrigel invasion assay of unsorted and sorted CD24^low^/CD44^+^ MCF-7 cells, respectively. Representative pictures of the formed tumorspheres (**b**, bottom) or the migrated cells in invasion assay (arrowheads) (**c**, bottom) are shown. The data are the means ± SEM (n = 3, *p < 0.05, **p < 0.01, Student’s *t*-test). **d** The viability of MCF-7 cells after AvIR-mediated PIT (3 J/cm^2^) with the indicated BioAb. The data are the means ± SEM (n = 3, **p < 0.01, Student’s *t*-test). **e** Clonogenicity of AvIR-PIT-treated surviving MCF-7 cells was evaluated by tumorsphere-formation assay. The data are the means ± SEM (n = 3, *p < 0.05, **p < 0.01, one-way ANOVA with Tukey’s test, NS; not significant)

In order to investigate an application of AvIR-PIT for better control of tumors, we next examined the CSC population as a therapeutic target. MCF-7 cells contained CD24^low^/CD44^high^ subpopulation, which was judged to have highly tumorigenic CSC-like property based on tumorsphere-formation assay and Matrigel invasion assay (Fig. 4a–c). AvIR-PIT with Bio-CD24 or Bio-CD44 markedly reduced the viability of MCF-7 cells (Fig. 4d). When the cells that survived the CD44- or CD24-targeted AvIR-PIT treatment were tested in tumorsphere assay, it was revealed that the sphere forming capacity of the PIT-treated cells was substantially abolished (Fig. 4e). Note that, although the observed phototoxicity for cell viability of AvIR-PIT with Bio-CD44 was lower than that of AvIR-PIT with Bio-CD24 (Fig. 4d), the anti-tumorigenic effect of the former was almost equal to the latter, suggesting superior effectiveness of CSC-targeting in PIT treatment on tumor control.

**Fig. 5:**
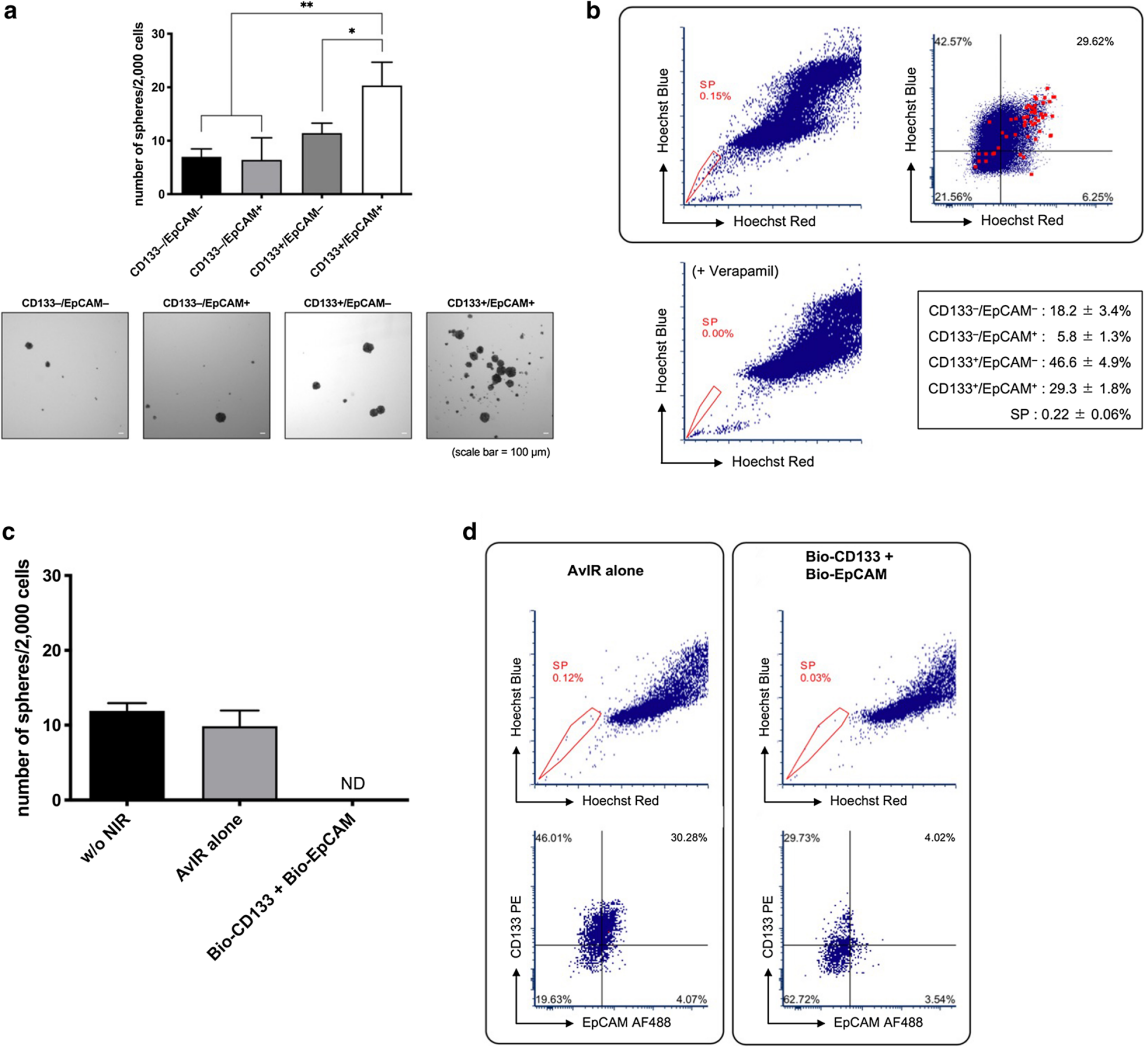
Effect on HuH-7 SP cells by AvIR-mediated PIT. **a** Clonogenicity of HuH-7 cells was evaluated by tumorsphere-formation assay. HuH-7 cells were sorted to CD133^−^/EpCAM^−^, CD133^−^/EpCAM^+^, CD133^+^/EpCAM^−^, or CD133^+^/EpCAM^+^ subpopulation. Using each subpopulation of HuH-7 cells, tumorsphere-formation assay was performed in PromoCell Cancer Stem Cell medium. The data are the means ± SEM (n = 3, *p < 0.05, **p < 0.01, one-way ANOVA with Tukey’s test). Representative images of tumorspheres are also shown in the bottom panels. **b** Identification of SP cells in HuH-7 cell line. HuH-7 cells were stained with Hoechst 33342 and analyzed by flow cytometry. The SP cells, which disappear in the presence of verapamil (50 µg/ml; bottom left panel), are outlined and shown as a percentage of the total cell population. Expression profile of CD133 and EpCAM in HuH-7 cells is shown in the top right panel, in which red dots indicates the SP cells by back-gating. The percentage of each subpopulation is also shown in the bottom right panel (means ± SEM, n = 4; bottom right panel). **c** Sphere-forming capability of AvIR-PIT-treated HuH-7 cells. By using Bio-CD133 and Bio-EpCAM, CSC-like subpopulation-targeted AvIR-PIT was performed against HuH-7 cells. After removal of the dead cells by using ClioCell magnetic particles, the resulting live cells were examined by tumorsphere-fomation assay. The data are the means ± SEM (n = 3, ND; not detected). **d** FACS analysis of AvIR-PIT-treated HuH-7 cells. The survived cells collection from CSC-targeted AvIR-PIT was performed as in **c**. The cells were further cultured by 2 passages and FACS-analyzed on SP and expression of CD133 and EpCAM

To further investigate the efficacy of CSC-targeted AvIR-PIT, we next used HuH-7 cell line, in which CD133^+^/EpCAM^+^ subpopulation was reported to have a CSC-like property [18]. Indeed, in our hands, the CD133^+^/EpCAM^+^ HuH-7 cells were capable of forming much more tumorspheres than the other population (Fig. 5a). We also found that the SP cells, which are defined by their ability to exclude the DNA-binding dye Hoechst 33342 and known to share some characteristics of CSCs [19], were highly enriched in the CD133^+^/EpCAM^+^ subpopulation (Fig. 5b). We performed tumorsphere assay using 2000 viable cells after AvIR-PIT with Bio-CD133 and Bio-EpCAM (2.5 µg/ml each) and found that the PIT treatment completely abolished the sphere-forming ability of HuH-7 cells (Fig. 5c). The survivors from the AvIR-PIT treatment were further subcultured for another 2 passages and then subjected to FACS analysis. It was revealed that CD133^+^/EpCAM^+^ cells that had been killed by AvIR-PIT with Bio-CD133 and Bio-EpCAM hardly reemerged after passaging and SP cells did little as well (Fig. 5d), suggesting that successful CSC-targeted killing was elicited and that CSC-targeted AvIR-PIT can effectively dampen the tumorigenicity of surviving HuH-7 cells.

### AvIR-PIT against the cells composing tumor microenvironment

**Fig. 6:**
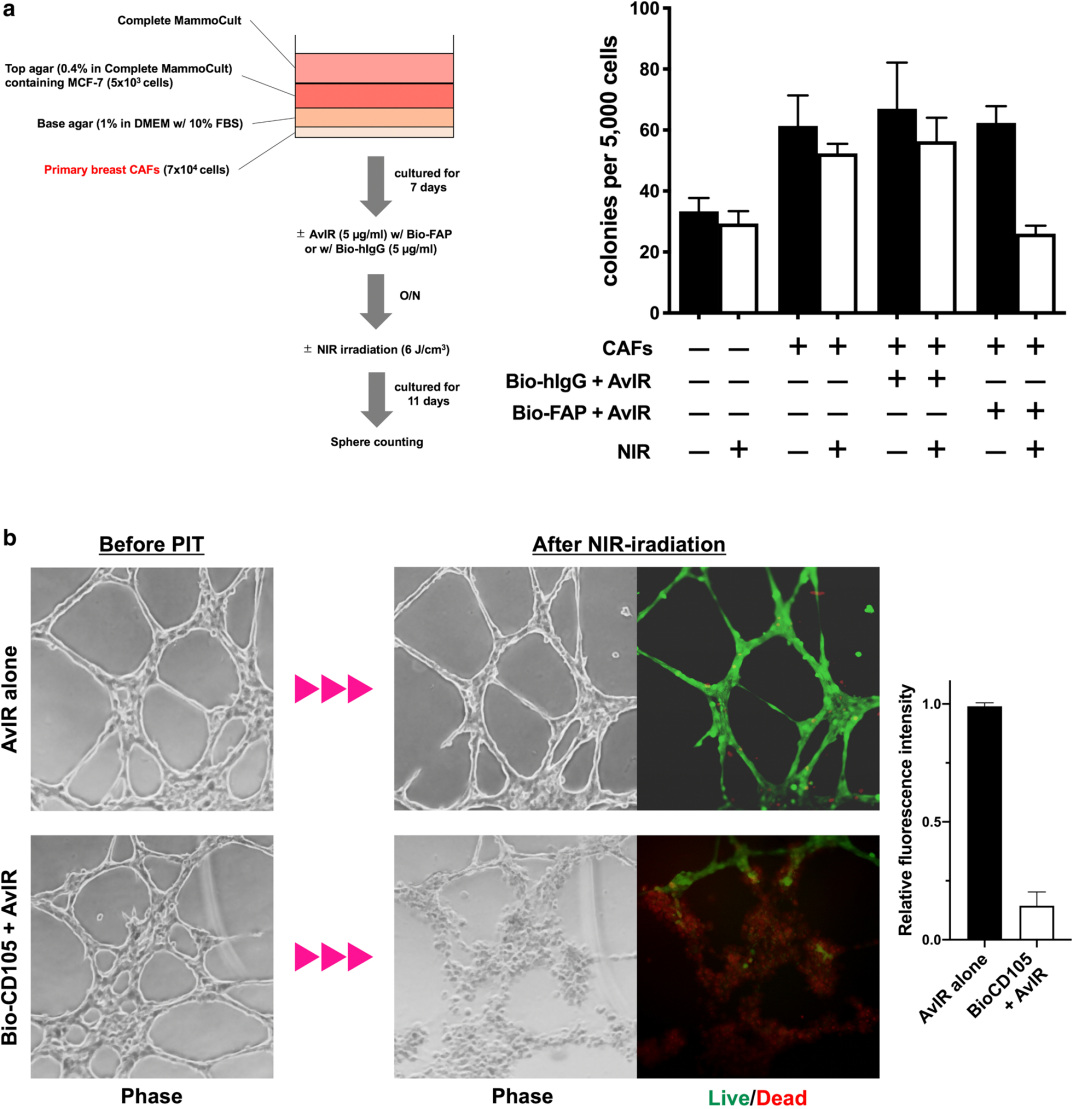
AvIR-mediated PIT targeting tumor microenvironment. **a** Effect on FAP-targeted AvIR-PIT on the clonogenicity of MCF-7 cells co-cultured with CAFs. The clonogenicity was evaluated by soft agar colony formation assay as described in Materials and methods. Schematic representation of the soft agar-culturing is shown in the left panel. The number of MCF-7 colonies was counted on 11 days after FAP-targeted AvIR-PIT treatment. The data are the means ± SEM (n = 3). **b** Effect on CD105-targeted AvIR-PIT on the capillary-like structure formed by 2H-11 tumor endothelial cells. LIVE/DEAD cell imaging was performed against the 2H-11 tubes after AvIR-mediated PIT with Bio-CD105. Data in the rightmost panel shows the relative fluorescence intensity, i.e. the ratio of total green fluorescence intensity per well after NIR irradiation to that of before the irradiation. The data are the means ± SEM (n = 3)

In order to further verify the applicability of AvIR-mediated PIT, non-malignant cells that construct tumor microenvironment were targeted. We first performed a modified soft agar colony assay with MCF-7 cells and human primary breast CAFs (Fig. 6a; see also “Materials and methods”). When MCF-7 cells in the top agar were co-cultured with CAFs at the bottom of the culture well, much more MCF-7 colonies were formed than when MCF-7 cells were cultured alone, indicating the capability of CAFs to accelerate the tumor cell clonogenicity (Fig. 6a). However, AvIR-PIT with BioAb against FAP, a specific marker of CAFs, was performed on culture day 8, the CAF-enhancing clonogenicity of MCF-7 was completely canceled. Next, AvIR-PIT with BioAb specific for CD105, one of the markers of tumor neovasculature, was performed against capillary-like tubular structures formed by tumor endothelial 2H-11 cells. As shown in Fig. 6b, the tube structures were collapsed by AvIR-PIT treatment using Bio-CD105, but not affected by AvIR-alone treatment with NIR irradiation. Taken together, these results demonstrate that AvIR-mediated PIT has great potential and versatility to kill not only tumor cells themselves but also various components of the tumor tissues effectively, if the targeting BioAbs are appropriately selected.

## Discussion

Tumors often have heterogeneous expression of surface antigens and may differ not only between individuals but even within the same patient [20]. Furthermore, tumor cells generally lose expression of the surface antigens during the progression of the malignancy, and such antigen loss is one of the major factors contributing to tumor relapse after specific therapy that was initially effective [21]. Thus, general antibody-based immunotherapy with a fixed target specificity cannot combat therapy-resistant cancers in many cases. Because of the marked target specificity and the localized NIR irradiation, if IR700-mAb conjugates specific to different TAAs depending on the situation were to be prepared each time, it would be possible to safely repeat the cancer-targeted PIT treatment without adverse side effects due to injury to normal tissues; however, it is unlikely to be practical. In this study, we provided a feasible and universal solution to such cumbersome circumstances by introducing the AvIR and BioAbs into PIT, while retaining the advantages of conventional one (Fig. 1). A notable merit of the AvIR-mediated PIT demonstrated here is that a wide variety of BioAbs are readily available and only avidin needs to be chemically conjugated to IR700 once prior to the PIT-treatment. Additionally, various biotinylated molecules such as biotin-labeled small compounds and nucleic acids may be potential candidates for cellular targeting ligands as like BioAbs, unless they are not internalized or incorporated into the target cells.

We and other group have previously shown that IR700-mediated PIT, in contrast to conventional photodynamic therapy, exerts the phototoxic effect as long as PS-conjugates bind to the target cell membrane without a need for their entries into the cell, and effectively works even in hypoxia because the phototoxicity results from the reduction of cell membrane integrity, which is induced by photochemical damage independent of production of reactive oxygen species [4, 5]. Such features are thought to be especially suitable for targeting the CSCs, because they have chemo-resistant property with enhanced drug excretion functions and reside in a hypoxic tumor niche [12, 19]. We demonstrated that AvIR-mediated PIT using BioAbs specific to CSC markers is able to reduce the tumorigenicity of MCF-7 and HuH-7 cell lines. Moreover, the viability of MKN-45 cells was greatly decreased by CD44-targeted AvIR-PIT (Fig. 3b). MKN-45 cell line has been reported to include tumorigenic CSC-like cells expressing stemness factors such as Oct4 and Sox2 in a CD44-positive subpopulation [22], thus suggesting that the CSC-like subpopulation of MKN-45 could also be effectively removed by the AvIR-PIT. As shown in Fig. 4, anti-tumorigenicity induced by AvIR-PIT with Bio-CD44 was as effective as with Bio-CD24, while the effect on the reduction of MCF-7 viability was weaker for Bio-CD44 than Bio-CD24. This implies that CSC-targeting strategy is sufficient to control tumor growth. However, considering the in vivo situation, because CSCs are hidden deep within tumors, it is likely to be important to kill bulk tumor cells (CD24^+^ cells in this case) and effectively deliver photosensitive agents to the site where CSCs exist. Therefore, simultaneous targeting of TAAs and CSC markers enabled by AvIR-mediated PIT would be useful for more efficacious tumor suppression and further for eradication of cancer cells. In antibody-based therapies, especially when mAbs with strong binding affinity are used and/or tumor cells express high levels of antigen, a phenomenon known as the “binding site barrier”, in which mAbs are saturated in the perivascular space and cannot penetrate deeper into the tumor, is sometimes problematic [23]. Nakajima et al. reported that the problem could be overcome by using a cocktail of two different IR700-mAbs for more homogeneous intratumoral distribution of the PIT agent, showing the enhanced therapeutic effects compared with the use of either IR700-mAb [24]. Such approach can also be readily applied to AvIR-based PIT by using BioAbs with different profiles.

We also demonstrated that AvIR-PIT can target the tumor-supportive cells that reside in tumor microenvironment, such as CAFs and the tumor endothelial cells. Because these types of cells have been shown to play a crucial role in the development and maintenance of the majority of solid tumors [25–27], the therapeutic approaches that target them are one of the non-limiting therapeutic strategies and can be effectively applied to the wide range of tumor types. In the light of clinical success of bevacizumab (Avastin^®^), a humanized mAb against vascular endothelial growth factor (VEGF), the anti-angiogenic treatment approaches of solid tumors have been extensively investigated, including a recent report by Nishimura and colleagues on tumor neovasculature-targeted PIT using IR700-conjugated anti-VEGF receptor 2 mAb [11]. Because CSCs are preferentially located in the specialized perivascular niche [28], which maintains stemness of them, and the disruption of the tumor vessels leads to increase in vascular permeability and leakage for macromolecules like BioAbs, combined treatment of CSCs and tumor vasculatures by AvIR-mediated PIT is supposed to be especially promising.

The application of PIT currently attracting the most attention is activation of tumor immunity. Previously, Sato et al. demonstrated that PIT with IR700-conjugated anti-CD25 for targeting regulatory T cells (T_regs_) can cause site-specific killing of T_regs_ in the NIR-irradiated tumor bed and induce regression of not only PIT-treated tumors but distant non-treated tumors [29]. This is probably due to locally activated CD8^+^ T cells and NK cells in the treated tumor site by spatially selective depletion of T_regs_ leading to reversal of immunosuppressive environment. If AvIR-mediated PIT is applied to this strategy, T_regs_ and/or other immune-suppressor cells, such as myeloid derived suppressor cells and tumor associated macrophages, can be surely targeted and treated simultaneously with tumor cells by using a cocktail of BioAbs against surface markers of the tumor cells and the suppressor cells. Combination with immune-checkpoint therapy may also be possible to further enhance the host immunity.

On the other hand, one of the important obstacles in successful clinical application of AvIR-PIT is the potential immunogenicity of NeutrAvidin to humans. However, this issue is likely to be avoidable by using, instead of NeutrAvidin, a hypoimmunogenic avidin mutant [30] or a Bradavidin II, which is originated from *Bradyrhizobium japonicum*, a nitrogen-fixing bacteria, and is reported to have low immunogenic potential [31], or more straightforwardly, by use of a commercially available humanized anti-biotin antibody. Another problem may be the molecular size of AvIR. NeutrAvidin, which was deglycosylated version of avidin and used for preparation of AvIR, contains four identical biotin-binding subunits with a total molecular mass of 60 kDa. This size is much smaller than that of IgG (~ 150 kDa) and close to that of immunoglobulin Fab fragment (~ 50 kDa). While such a small targeting protein might be undesirable for PIT due to the pharmacokinetics, i.e. faster clearance from the circulation and lower tumor retention, it is also likely to be superior in terms of rapid tumor accumulation and better penetration into tumor tissues. Actually, previous reports demonstrated the smaller antibody fragments are advantageous in some PIT settings [24, 32].

## Conclusions

In summary, we developed a novel type of PIT utilizing an AvIR, the IR700-conjugated avidin protein, as a universal PIT agent, together with BioAbs for specific cellular targeting. Our results suggest that AvIR-mediated PIT would enable a sequential or simultaneous targeting to not only bulk tumor cells but to multiple tumor-supporting and/or immunosuppressor cells and allow integrative and efficacious control of tumor and its microenvironment, overcoming the tumor heterogeneity. In vivo studies are now being pursued in our laboratory to further confirm the therapeutic potential and evaluate the clinical impact of AvIR-mediated PIT.

## Data Availability

All data generated or analyzed during this study are included in this published article.

## Abbreviations

ANOVA: analysis of variance
AvIR: IRDye700DX-conjugated NeutrAvidin
BioAb: biotinylated antibody
CAF: cancer-associated fibroblast
CEA: carcinoembryonic antigen
CSC: cancer stem cell
DMEM: Dulbecco’s minimal essential medium
EGFR: epidermal growth factor receptor
FACS: fluorescence-activated cell sorting
FAP: fibroblast activation protein
FBS: fetal bovine serum
FITC: fluorescein isothiocyanate
HBSS: Hanks balanced salt solution
HEPES: 4-(2-hydroxyethyl)-1-piperazineethanesulfonic acid
HER2: human epidermal growth factor receptor 2
IR700: IRDye700DX
mAb: monoclonal antibody
MEM: minimum essential medium
NIR: near infrared
PE: phycoerythrin
PIT: photoimmunotherapy
PS: photosensitizer
T_reg_: regulatory T cell
SEM: standard error of the means
SP: side-population
TAA: tumor-associated antigen
VGEF: vascular endothelial growth factor
